# LncRNA H19‐Encoded Micropeptide altH19 Promotes DNA Replication and Mitosis in Myeloma Cells by Enhancing the Phosphorylation of CDK2 at Threonine 160

**DOI:** 10.1111/cpr.70089

**Published:** 2025-06-27

**Authors:** Yaxin Zhang, Wenjing Li, Xu Cao, Jiwei Mao, Xiaodan Zhou, Linlin Liu, Ruosi Yao

**Affiliations:** ^1^ Department of Hematology The Affiliated Hospital of Xuzhou Medical University Xuzhou China; ^2^ Blood Diseases Institute Xuzhou Medical University Xuzhou China; ^3^ College of Medical Imaging Xuzhou Medical University Xuzhou China

**Keywords:** CDK2, DNA replication, micropeptide, mitosis, multiple myeloma

## Abstract

Micropeptides are endogenous peptides translated from alternative open reading frames (alt‐ORFs) within coding or non‐coding genes. Emerging evidence suggests that some micropeptides play critical roles in both physiological and pathological processes. Multiple myeloma (MM), a haematological malignancy, remains incurable due to frequent relapses and a limited understanding of its underlying mechanisms. In this study, we sought to investigate the function and molecular mechanism of a novel micropeptide in MM pathogenesis. We identified a novel micropeptide, altH19, encoded by the lncRNA H19, which is highly expressed in patients of MM. Functional assays revealed that altH19 promotes myeloma cell proliferation and colony formation significantly. Furthermore, altH19 induces multipolar mitosis by upregulating the expression of Aurora B, Centrin 2 and phosphorylated histone H3. Flow cytometry analyses confirmed that overexpression of altH19 enhances DNA replication and accelerates the transition from early to mid‐late stages of the DNA replication process. Conversely, knockout of altH19 reverses these effects. Mechanistically, altH19 directly interacts with phosphorylated CDK2 at threonine 160, thereby enhancing CDK2 T160 phosphorylation and activating the downstream E2F1 target RB phosphorylation. Notably, altH19 was able to restore phosphorylation levels of CDK2 and RB that were otherwise suppressed by the CDK2‐selective inhibitor Seliciclib. In summary, we identify altH19 as a novel lncRNA‐derived micropeptide with a pivotal role in myeloma progression, highlighting the therapeutic potential of targeting the altH19‐CDK2‐RB axis in MM treatment.

## Graphical abstract

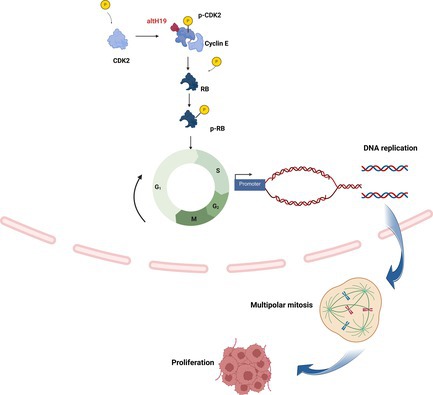

## 1 Introduction

Micropeptides are a class of endogenous peptides translated from small open reading frames (sORFs) or alternative open reading frames (altORFs), which are often evolutionarily conserved [1]. The first functional microprotein was identified in 1990 by Benezra et al. [2], who discovered a novel helix–loop–helix protein known as the ‘Id’ protein modulating muscle differentiation. Recent advances have revealed that many sORFs are actively translated in regions previously annotated as non‐coding, including untranslated regions (UTRs) of mRNAs and various lncRNAs [3]. Functionally, micropeptides have been implicated in a broad range of biological processes, such as RNA processing, DNA repair, metabolism, muscle development and drug resistance [4, 5, 6, 7, 8]. Furthermore, a growing number of micropeptides have been linked to disease progression, including breast cancer, hepatocellular carcinoma, glioblastoma, colorectal cancer, melanoma and acute myeloid leukaemia [9, 10, 11, 12, 13, 14]. In our previous work, we identified a novel micropeptide altKLF4 derived from the transcription factor KLF4, which plays a role in modulating chemotherapeutic sensitivity [15]. Despite these advances, the mechanistic role of micropeptides in the development of multiple myeloma (MM) remains largely unexplored.

MM is a malignant haematological disease that remains incurable, primarily due to drug resistance and frequent relapse [16]. Despite advances in therapeutic strategies, the molecular mechanisms underlying MM progression and development are still not fully understood. Previous studies have largely focused on aspects such as epigenetic regulation, the tumour microenvironment, multidrug resistance and genetic abnormalities [17, 18, 19, 20]. Among these, genetic abnormalities represent a critical driver of MM progression, with common abnormalities including hyperdiploidy and chromosomal translocations such as t(4;14), t(11;14), gain(1q) and del(17p) [21]. Notably, chromosomal instability (CIN) is considered a major inducer of genetic lesions, ultimately contributing to MM initiation and evolution. Cyclin‐dependent kinase 2 (CDK2) is a key regulator of cell cycle progression, specifically facilitating the transition from the G1 to S phase and the progression through S phase by forming complexes with Cyclin E and Cyclin A [22]. The CDK2/Cyclin E complex is frequently hyperactivated in various human cancers and is associated with poor prognosis, due to its essential role in DNA replication. Furthermore, oncogenic activation of CDK2 has been shown to induce genomic instability in multiple model systems [23]. Based on this, we aim to elucidate the mechanisms through which chromosomal instability contributes to MM pathogenesis, with a particular focus on the role of CDK2.

LncRNA H19 gene is one of the earliest identified imprinted genes and is highly conserved across evolution. It has been implicated in the progression of various cancers [24]. Notably, elevated expression of H19 is significantly associated with poor prognosis in MM [25]. In this study, we identify a previously unrecognised open reading frame (ORF) within the H19 gene that encodes a 97‐amino acid polypeptide, which we have designated as altH19. Our findings reveal that overexpression of altH19 markedly promotes the proliferation of MM cells, while CRISPR‐mediated knockout of altH19 significantly impairs cell proliferation. Additionally, we observed that altH19 accelerates DNA replication and mitotic progression in MM cells. Mechanistically, altH19 interacts with phosphorylated CDK2 at threonine 160 (p‐CDK2 T160), enhancing its phosphorylation activity. This, in turn, activates the downstream E2F target pathway, particularly RB phosphorylation, thereby promoting DNA replication efficiency and mitotic entry. In summary, our study identifies altH19 as a novel lncRNA‐derived micropeptide with a critical role in the regulation of DNA replication and mitosis. The discovery of altH19 provides new mechanistic insights into MM pathogenesis and suggests potential therapeutic avenues targeting the altH19‐CDK2‐RB axis.

## 2 Materials and Methods

### 2.1 GEO Dataset Analysis and Sample Collection

Publicly available datasets GSE2658 and MMRF‐COMMPASS (*n* = 831) are obtained from the GEO database [26] and MMRF Researcher Gateway, respectively. Additionally, this study included bone marrow samples from 14 newly diagnosed MM patients and 14 healthy donors, recruited from the Affiliated Hospital of Xuzhou Medical University. Written informed consent was obtained from all participants prior to sample collection. All procedures involving human subjects were approved by the Ethics Committee of the Affiliated Hospital of Xuzhou Medical University, in accordance with institutional and national guidelines.

### 2.2 Cells and Reagents

HEK‐293T and MM cell RPMI‐8226 and U266 were cultured in DMEM medium and 1640 medium supplemented with 10% ZETA serum, respectively. All cells were maintained in a humidified incubator at 37°C with 5% CO_2_. The polyclonal antibody against altH19 was synthesised by Dian Biotechnology (Wuhan, China). The CDK2 inhibitor Seliciclib was purchased from TargetMol (shanghai, China). The following antibodies were used in this study: GAPDH (60004‐1‐Ig), CDK2 (10122‐1‐AP), RB (10048‐2‐Ig), Centrin 2 (15877‐1‐AP), Aurora B (39261), Cyclin B1 (55004‐1‐AP) and CDK1 (19532‐1‐AP) were obtained from Proteintech (Wuhan, China). The Flag antibody (RA1003‐01) was purchased from Vazyme Biotech (Nanjing, China). Antibodies against phospho‐histone H3 (p‐H3, AF3358), phospho‐RB (Ser780) (p‐RB, AF3103) and phospho‐CDK2 (Thr160) (p‐CDK2, AF3237) were obtained from Affinity Biosciences.

### 2.3 Cell Proliferation Assays

For CCK‐8 assay, 5000 cells per well were seeded in a 96‐well plate. At specified time points (0, 12, 24, 36 and 48 h), 5 μL of CCK‐8 reagent was added to each well and incubated at 37°C for 2.5 h. Then absorbance was measured using a microplate reader. For colony formation assay, 1000 cells per well were seeded in six‐well plate with 2 mL methylcellulose‐containing RPMI‐1640 medium. After 14 days of incubation at 37°C, 200 μL of Wright‐Giemsa stain was added to each well and incubated overnight at 4°C. Colonies were subsequently photographed and counted. For EdU incorporation assay, 1 × 10^5^ cells per well were seeded in six‐well plate and treated with 10 μM EdU for 2 h. Then cells were harvested and incubated with 300 μL reaction solution. The percentage of EdU‐positive cells was analysed via flow cytometry.

### 2.4 Cell Synchronisation Treatment

RPMI‐8226 and U266 cells were synchronised with 2.5 μM thymidine (TdR) for 24 h. Then cells were washed and cultured in complete RPMI‐1640 medium for 6 h. Subsequently, cells were re‐treated with 2.5 μM TdR for another 24 h. After synchronisation, the cell cycle distribution was analysed using flow cytometry.

### 2.5 Western Blot Analysis

Following cell synchronisation, indicated cells were lysed using RIPA lysis buffer (Beyotime, Shanghai, China). Protein samples were mixed with 2 x loading buffer, then separated by SDS‐PAGE. The resolved proteins were transferred to PVDF membranes, which were subsequently blocked in 5% BSA in PBST for 1 h. The membranes were incubated with primary antibodies followed by HRP‐conjugated secondary antibodies. Finally, the protein bands were visualised using ECL reagents.

### 2.6 Immunofluorescence (IF) Assay

Synchronised cells were fixed with 4% paraformaldehyde at room temperature. Then cells were incubated with primary antibodies (α‐tubulin, Phalloidin, altH19 and p‐CDK2, 1:100) at 37°C for 2 h. After washing three times with PBST, fluorescently labelled secondary antibodies were added and incubated at 37°C for 1 h. Then the slides were washed three times with PBST and stained with DAPI. Lastly, fluorescence was observed using a Zeiss LSM880 confocal laser scanning microscope.

### 2.7 Flow Cytometry Assay

After cell synchronisation, RPMI‐8226 and U266 cells were collected and fixed in ethanol, followed by overnight incubation at 4°C. Then cells were incubated with primary antibodies against P21, Cyclin D1, Cyclin E1 and Ki67 at 37°C for 1 h. After three washes with PBS, cells were incubated with corresponding fluorescent secondary antibodies at 37°C for 1 h. Flow cytometry was then performed to measure protein expression. For cell cycle analysis, cells were stained with propidium iodide (PI) in place of antibodies. Similarly, the cells were incubated with Ki67 and PI simultaneously at 37°C for 1 h, followed by flow cytometry analysis to assess relative DNA content.

### 2.8 DNA Replication Rate Detection Assay

After dual synchronisation, cells were incubated with EdU at 37°C for 2 h. The click reaction was performed according the EdU staining kit, and EdU incorporation was observed under a fluorescence microscope. Cells in replication phase and those transitioning to middle‐to‐late stages were counted separately.

### 2.9 Co‐Immunoprecipitation (Co‐IP) Assay

Briefly, indicated cells were lysed in NP‐40 lysis buffer and whole protein extracts were incubated with 2 μg of CDK2, altH19 or p‐CDK2 antibody at 4°C overnight with gentle rotation. Then 20 μL agarose beads were added and incubated for another 4 h at 4°C. The supernatant was discarded and agarose beads were washed twice with PBS. Lastly, the samples were mixed with 2× loading buffer and denatured, followed by western blot analysis.

### 2.10 Protein Purification and GST Pull Down Assay

pGEX‐6P‐1‐CDK2 plasmid was transformed into *Escherichia coli* BL21 cells and grown at 37°C for 6 h. Protein expression was induced with 0.1 mM IPTG for 8 h at 25°C. Bacterial cells were lysed via sonication and centrifuged at 12 000 rpm. The supernatant was incubated with GST magnetic beads at 4°C for 6 h with gentle rotation. CDK2 protein was eluted using reduced glutathione.

For GST pull down assay, GST magnetic beads were incubated with GST‐CDK2 protein at 4°C for 2 h. Flag‐tagged altH19 protein was then added to the bead‐CDK2 mixture and incubated at 4°C for 2 h. After discarding the supernatant via centifugation, the beads were washed three times with PBS, and the protein interactions were verified by western blot.

### 2.11 Statistical Analysis

Data are presented as mean ± standard deviation (SD). Student's *t*‐test was used for two‐group comparisons, and one‐way ANOVA was applied for multiple‐group comparisons. Data analysis was performed using GraphPad Prism 5 (GraphPad Software Inc., San Diego, CA). *p* < 0.05 was considered statistically significant.

## 3 Results

### 3.1 LncRNA H19 Encodes a 97‐AA Micropeptide Named altH19

**FIGURE 1:**
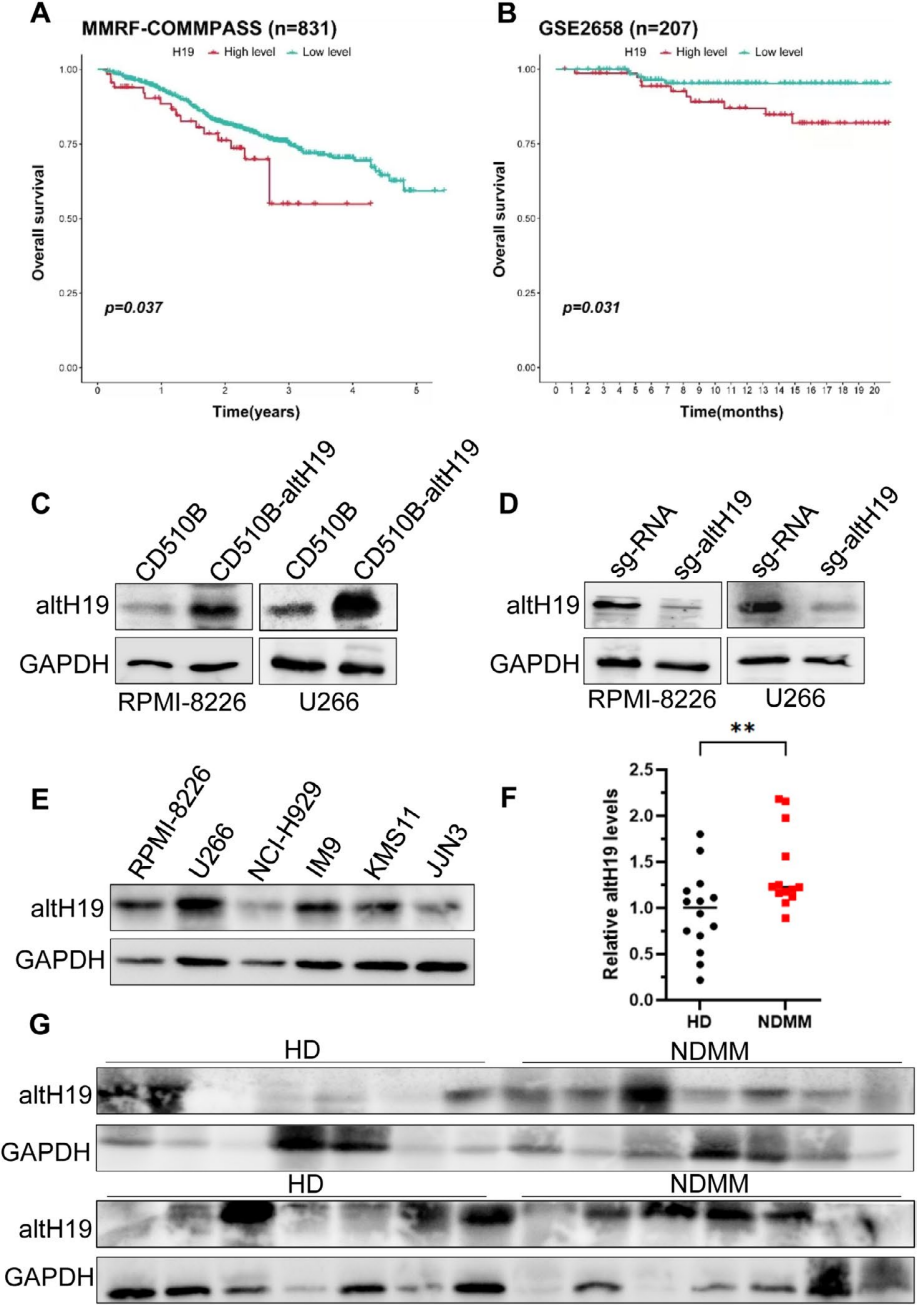
LncRNA H19 encodes a micropeptide. (A, B) Datasets from MMRF‐COMMPASS and GSE2658 showing the correlation between H19 expression and overall survival rate. (C) Western blot analysis of altH19 expression in altH19‐overexpressing and CD510B control RPMI‐8226 and U266 cells. (D) Western blot analysis of altH19 knockout efficiency in RPMI‐8226 and U266 cells. (E) Western blot analysis of altH19 differential expression in various myeloma cell lines. (F, G) Western blot analysis of altH19 protein expression in14 healthy donors (HD) and 14 newly diagnosed multiple myeloma (NDMM) samples, with scatter plot indicating differential expression. **p < 0.01.

In our previous transcriptome sequencing study, we identified several novel unannotated transcripts and proteins. Among these, a 97‐amino acid polypeptide sequence captured our attention, which might represent an unknown protein through protein sequence alignment and showed significant homology with partial sequences from multiple species (Figure S1A). Further analysis of the gene sequence corresponding to this polypeptide revealed that its transcript was located at the 5′ end of lncRNA H19 (Figure S1B). Interestingly, its parent gene H19 was strongly associated with poor prognosis in MM patients based on data from MMRF‐COMMPASS and GSE2658 databases (Figure 1A,B). We speculated that this micropeptide could be an altORF of lncRNA H19 and therefore named it altH19. To further confirm the protein‐coding potential of altH19, we designed and synthesised the altH19 gene, which includes a fragment of H19 5′ end before transcription initiation site (ATG) and a Flag tag before the stop codon (TGA) (Figure S1C). This construct was transfected into HEK‐293T cells, and western blot confirmed that it indeed encoded a protein with a molecular weight of approximately 10 kDa (Figure S1D). Then we developed a specific polyclonal antibody targeting altH19 and assessed its expression in RPMI‐8226 and U266 cells with stable overexpression and knockout of altH19 (Figure 1C,D). We also observed differential expression of the altH19 micropeptide across various MM cell lines (Figure 1E). Additionally, we measured altH19 expression levels in bone marrow plasma samples from healthy individuals and MM patients. The results showed that MM patients exhibited significantly higher altH19 protein levels in their bone marrow plasma (Figure 1F,G), suggesting that altH19 plays an important role in myeloma progression.

### 3.2 altH19 Promotes Multiple Myeloma Cell Proliferation

**FIGURE 2:**
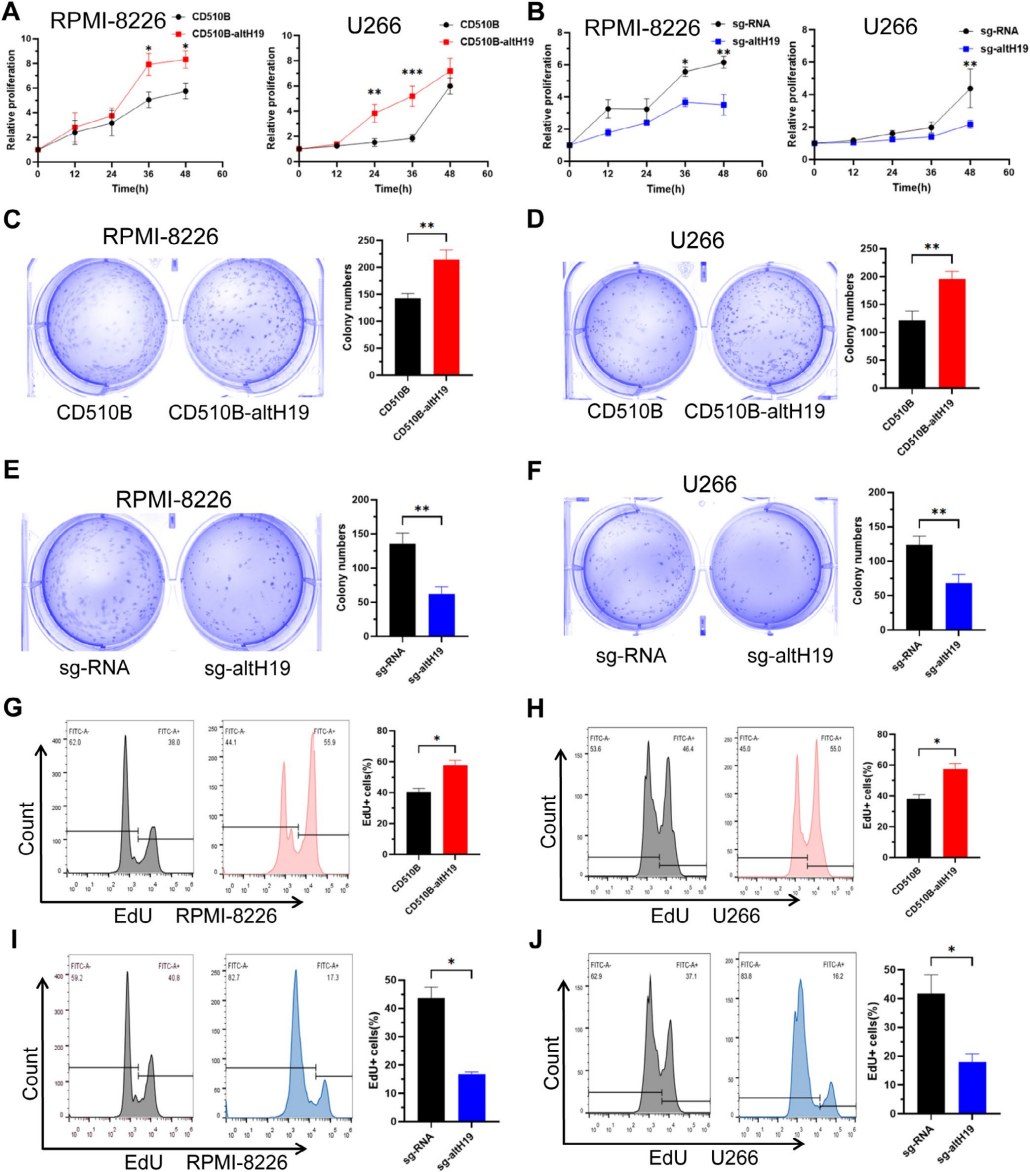
altH19 promotes myeloma cell proliferation. (A) CCK8 analysis of cell viabilities in RPMI‐8226 and U266 cells overexpressing altH19 at different time points. (B) CCK8 analysis of cell viabilities in RPMI‐8226 and U266 cells with altH19 knockout at different time points. (C, D) Colony formation assay showing the effect of altH19 overexpression on colony numbers in RPMI‐8226 and U266 cells. (E, F) Colony formation assay indicating the effect of altH19 knockout on colony numbers in RPMI‐8226 and U266 cells. (G, H) Flow cytometry analysis of EdU‐positive cells in RPMI‐8226 and U266 cells overexpressing altH19. (I, J) The effect of altH19 knockout on EdU incorporation in RPMI‐8226 and U266 cells. Experiments were performed at least three times. Mean ± SD. *p < 0.05; **p < 0.01; ***p < 0.001.

To investigate the function of altH19, we performed transcriptome sequencing on both wild‐type RPMI‐8226 cells and altH19‐overexpressing cell lines. Analysis of the sequencing data revealed that altH19 primarily regulated microtubule polymerisation and DNA replication in MM cells (Figure S1E). Based on these findings, we first explored the impact of altH19 on MM cell proliferation without double synchronisation using thymidine. CCK‐8 assay showed that RPMI‐8226 and U266 cell lines overexpressing altH19 exhibited significantly higher proliferation rates compared to wild‐type cells (Figure 2A). In contrast, altH19‐knockout cell lines demonstrated relatively slower proliferation (Figure 2B). Additionally, colony formation analysis revealed that the altH19‐overexpressing cell lines formed significantly more colonies than wild‐type cells (Figure 2C,D). Conversely, altH19 knockout resulted in a substantial reduction in the number of colonies compared to wild‐type cells (Figure 2E,F). Furthermore, EdU assay indicated that altH19 overexpression increased the proportion of MM cells in the proliferative phase (Figure 2G,H), while altH19 knockout reduced this proportion significantly (Figure 2I,J). These findings demonstrate that altH19 effectively promotes the proliferation of MM cells.

### 3.3 altH19 Induces Mitosis in Myeloma Cells

Cell proliferation is tightly regulated by the cell cycle. To investigate the role of altH19 in cell cycle progression, we first performed double synchronisation treatment using thymidine on wild‐type, altH19‐overexpressing and altH19‐knockout cells at the G1 phase (Figure S2A). Flow cytometry analysis revealed that altH19 overexpression increased the proportion of cells in the G2 phase (Figure S2B,C), while altH19 knockout resulted in a high proportion of cells in the G1 and S phases (Figure S2D,E). These findings suggest that altH19 promotes rapid division of myeloma cells. It has been reported that cyclin B1 is predominantly expressed during the G2/M phase of cell cycle and plays a key role in cell mitosis progression. Cyclin B1 typically forms a complex with CDK1 to initiate the early events of mitosis [27]. To assess the impact of altH19 on these regulators, we examined the expression of cyclin B1 and CDK1 in our experimental system. Western blot analysis showed that altH19 overexpression increased the levels of cyclin B1 and CDK1 significantly, whereas knockout of altH19 reversed this effect in both RPMI‐8226 and U266 cells (Figure S2F,G). These results suggest that altH19 regulates the key stages of cell cycle, particularly mitosis.

**FIGURE 3:**
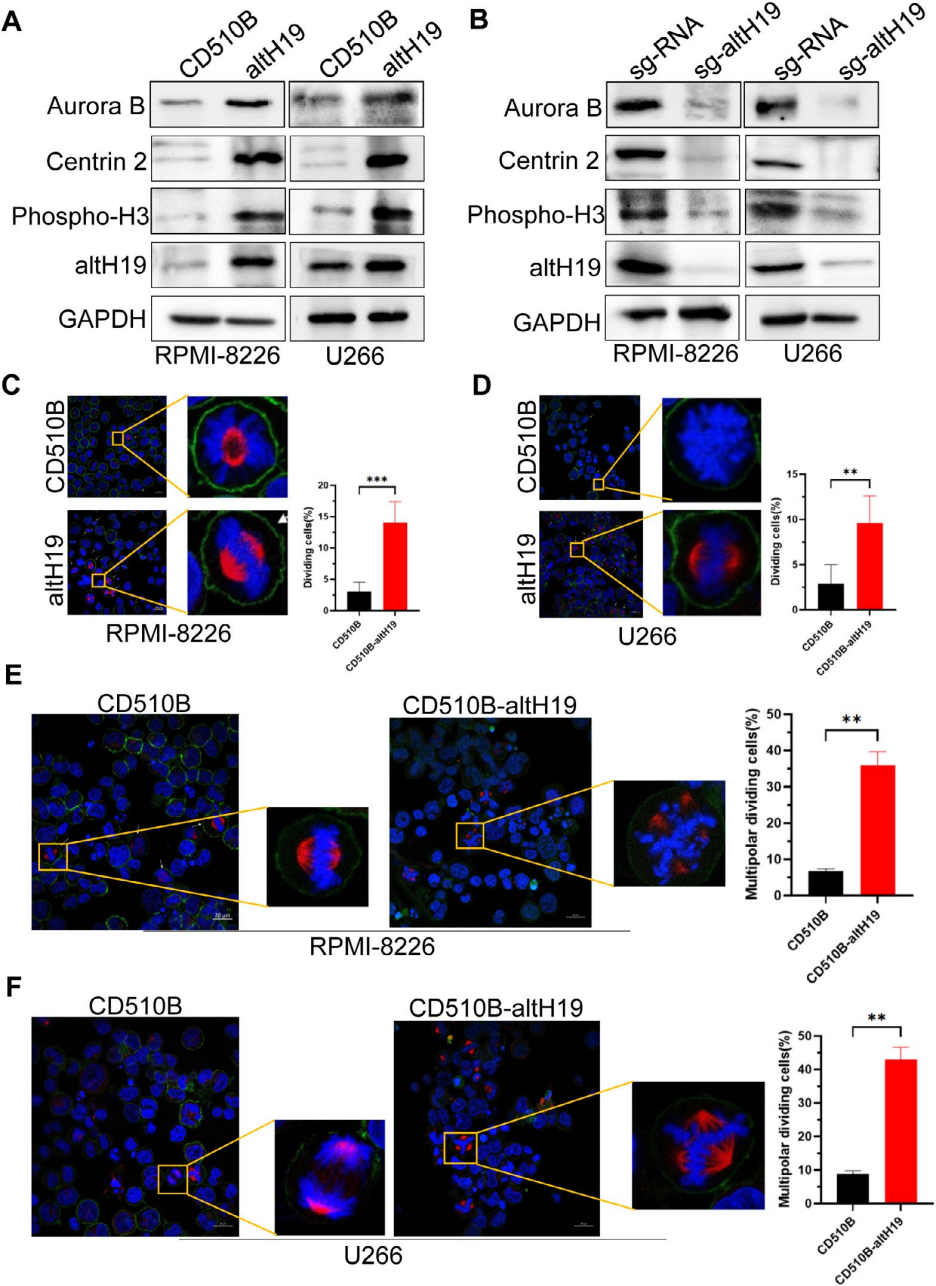
altH19 induces mitosis in myeloma cells. (A) Western blot analysis of Aurora B, Centrin 2, phospho‐H3 and altH19 expression in altH19‐overexpressing cells. (B) Western blot analysis of Aurora B, Centrin 2 phospho‐H3 and altH19 expression in altH19‐knockout cells. (C, D) Immunofluorescence staining was conducted using α‐tubulin, phalloidin and DAPI, followed by confocal microscopy analysis. The histogram indicates the number of dividing cells. (E, F) Confocal microscopy analysis of multipolar cell division, with a histogram showing the ratio of multipolar dividing cells. Data were presented as mean ± SD from three independent experiments. **p < 0.01; ***p < 0.001.

To further confirm this, we performed western blot analysis to detect centrosome‐associated proteins in TdR‐synchronised cells. The results demonstrated that altH19 overexpression led to a significant upregulation of Aurora B, Centrin 2 and phospho‐H3 proteins (Figure 3A), while altH19 knockout resulted in a marked decrease in the expression of these proteins (Figure 3B). These findings indicate that altH19 strongly promotes mitosis in myeloma cells. Additionally, immunofluorescence staining using α‐tubulin, phalloidin and DAPI, followed by confocal microscopy, revealed that the proportion of mitotic cells was significantly higher in the altH19‐overexpressing group compared to the control group (Figure 3C,D). We also conducted a statistical analysis of multipolar mitosis induced by altH19, which showed a high incidence of multipolar divisions in altH19 overexpression group (Figure 3E,F). This suggests the possible presence of abnormal DNA synthesis or an increased replication rate. Together, these findings imply that altH19 plays a crucial role in regulating mitotic progression in MM cells by enhancing DNA replication.

### 3.4 altH19 Promotes MM Cell DNA Replication

**FIGURE 4:**
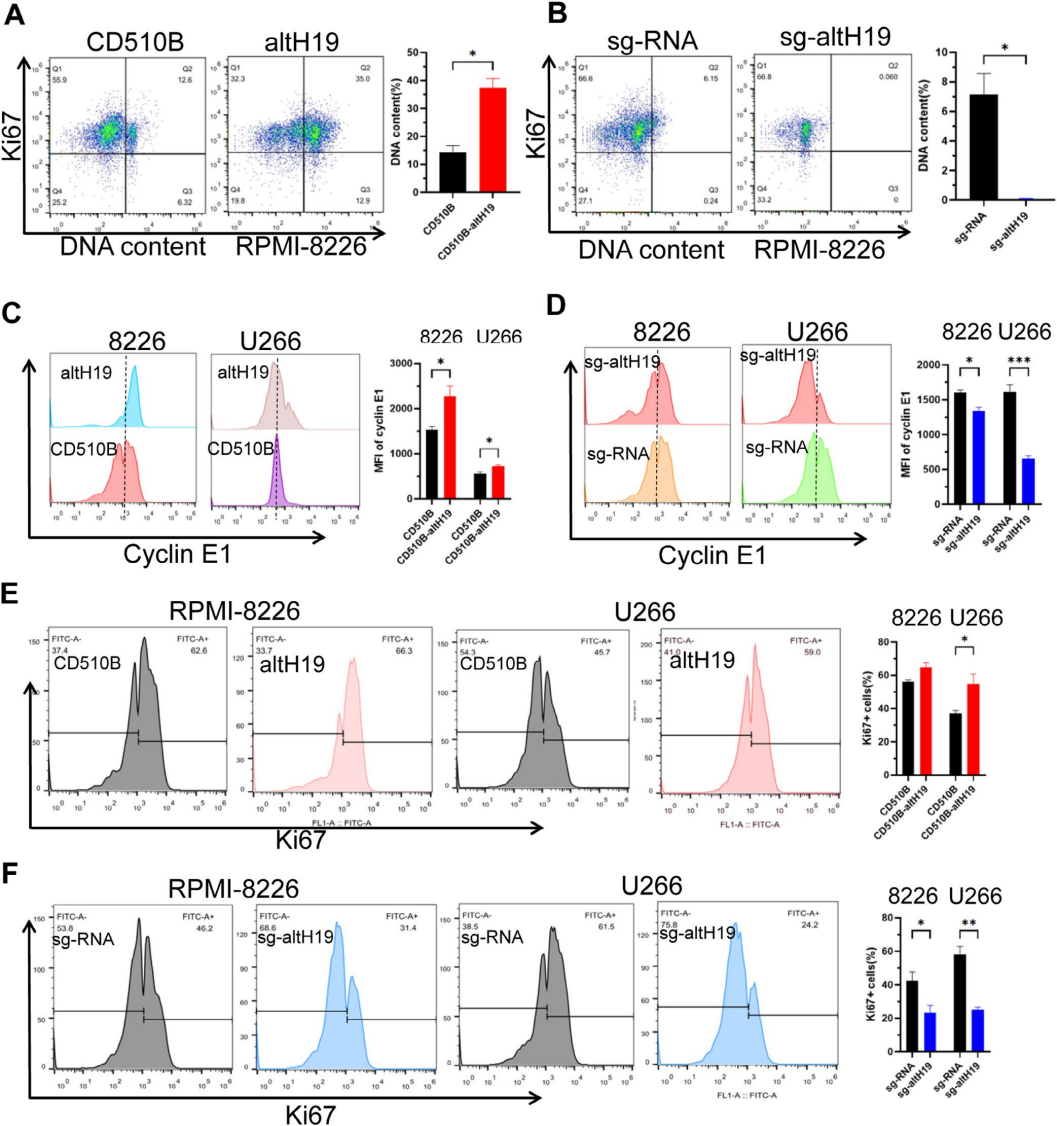
altH19 promotes MM cell DNA replication. (A, B) Flow cytometry analysis of Ki67 and PI dual staining in altH19‐overexpressing or knockout RPMI‐8226 cells. (C, D) Flow cytometry analysis of cyclin E1 expression, with mean fluorescent intensity in altH19‐overexpressing or knockout RPMI‐8226 and U266 cells. (E, F) Flow cytometry analysis of Ki67‐positive cells in altH19‐overexpressing or knockout RPMI‐8226 and U266 cells. Data were analysed with three independent experiments. Mean ± SD. *p < 0.05; **p < 0.01; ***p < 0.001.

To further investigate the positive regulatory effect of altH19 on DNA replication, we performed TdR‐based double synchronisation treatment on various cell lines and assessed DNA content using Ki67 and PI dual staining. The results showed that DNA content was significantly higher in the altH19‐overexpressing cell lines (Figure 4A), whereas it was markedly reduced in the altH19‐knockout cell lines (Figure 4B). These findings further support the role of altH19 in promoting DNA replication. Next, we used flow cytometry to analyse the expression of key biomarkers associated with DNA replication. The results demonstrated that the positive regulator cyclin E1 was upregulated in altH19‐overexpressing cell lines compared to the control group (Figure 4C), while its expression was downregulated in the altH19‐knockout cell lines (Figure 4D). Similarly, the proportion of Ki67‐positive cells was higher in the altH19 overexpression group (Figure 4E), but significantly lower in the altH19 knockout group (Figure 4F). On the other hand, the negative regulators of DNA replication, P21 and cyclin D1, exhibited reduced expression in the altH19‐overexpressing cells (Figure S3A,B), but increased expression in the altH19‐knockout cells (Figure S3C,D). These findings indicate that altH19 facilitates DNA replication by modulating the expression of key cell cycle regulators.

**FIGURE 5:**
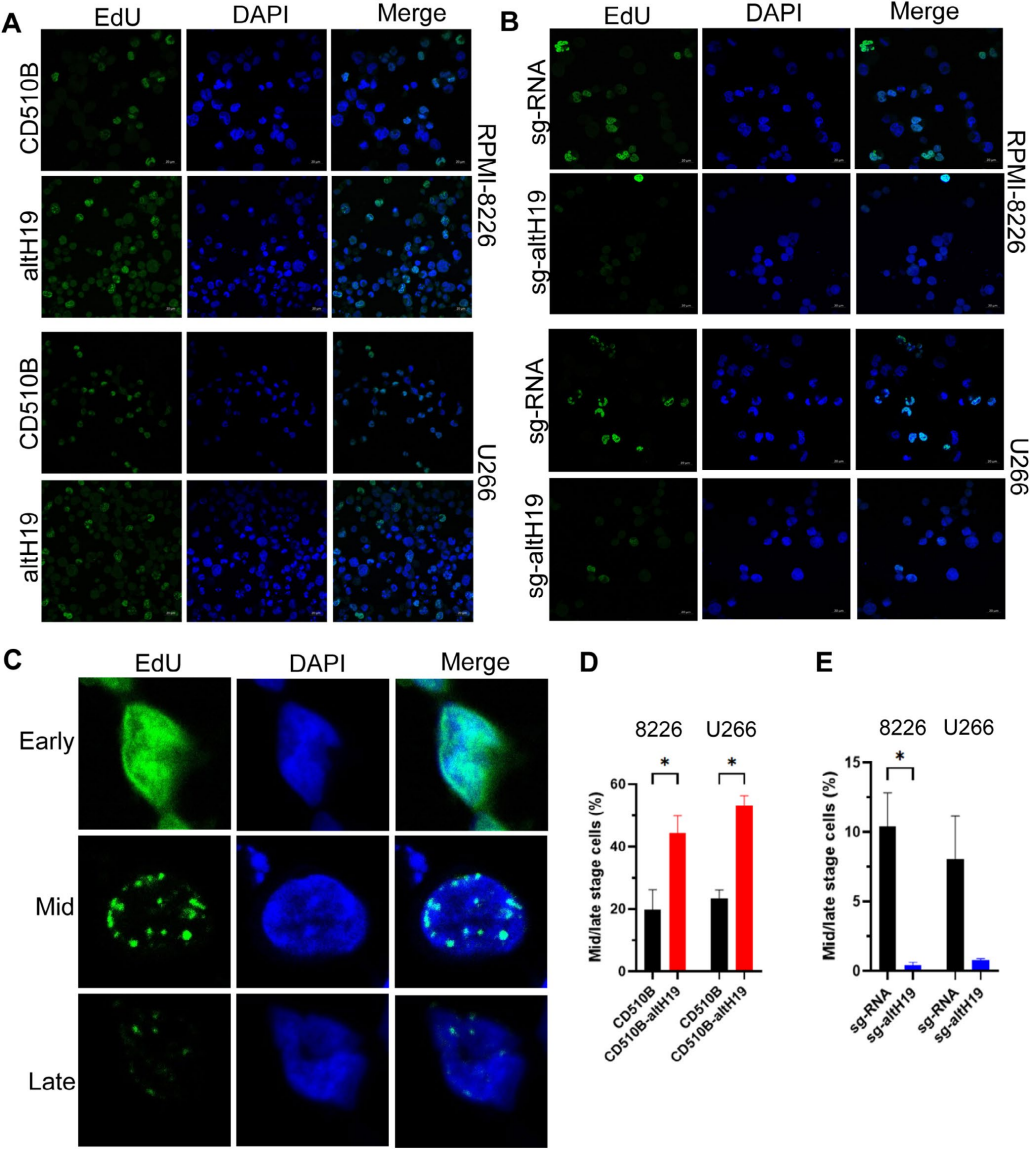
altH19 facilitates the proportion of cells in mid‐to‐late stages of DNA replication. (A) Immunofluorescence analysis of DNA replication progression using EdU staining in altH19‐overexpressing cells after synchronisation. (B) Immunofluorescence analysis of DNA replication progression using EdU staining in altH19‐knockout cells after synchronisation. (C) Representative images showing cells in mid‐to‐late stages of DNA replication. (D) Histogram indicating the percentage of cells in mid‐to‐late DNA replication stages overexpressing altH19. (E) Histogram analysis of mid‐to‐late stage cells in RPMI‐8226 and U266 cells after altH19 knockout. Experiments were repeated at least three times. Mean ± SD. *p < 0.05.

To further investigate the specific role of altH19 in DNA replication, cells were synchronised and incubated with EdU. Immunofluorescence analysis was then performed to examine the progression of DNA replication (Figure 5A,B). Statistical analysis revealed that altH19 overexpression increased the proportion of cells entering the DNA replication phase (Figure S3E), while altH19 knockout decreased the proportion of cells in DNA replication phase (Figure S3F). Additionally, the various stages of DNA replication were analysed using confocal microscopy (Figure 5C). A significant increase in cells at the mid‐to‐late stages of DNA replication was observed in altH19‐overexpressing group (Figure 5D). In contrast, knockout of altH19 disturbed the proportion of cells at these mid‐to‐late stages (Figure 5E). Taken together, these results suggest that the micropeptide altH19 accelerates DNA replication speed and facilitates the transition from early to mid‐late replication stages.

### 3.5 altH19 Binds to Phosphorylated CDK2 Directly

**FIGURE 6:**
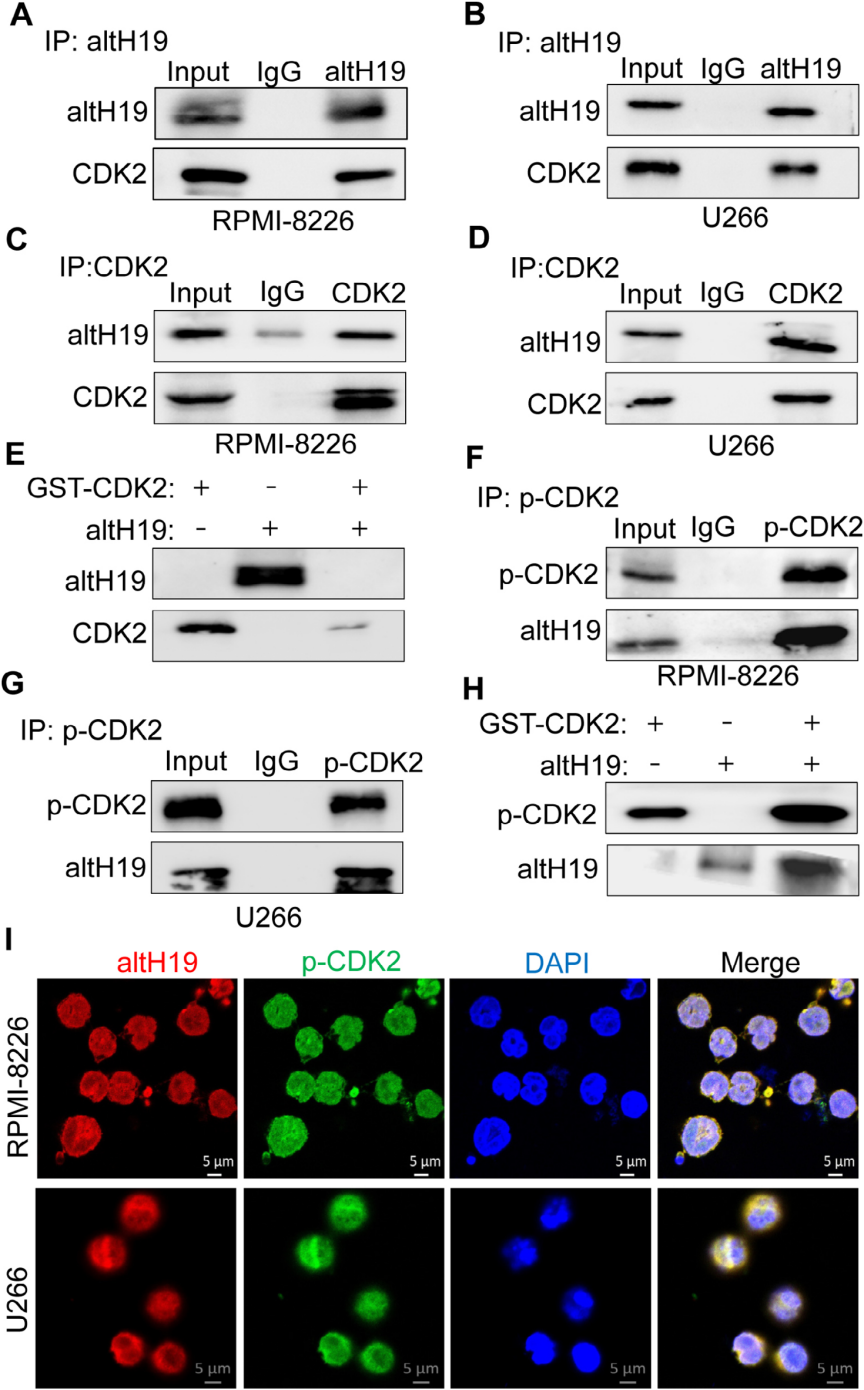
altH19 binds to phosphorylated CDK2 directly. (A, B) Immunoprecipitation using altH19 antibody in RPMI‐8226 and U266 cells, followed by western blot analysis of altH19 and CDK2 binding. (C, D) Immunoprecipitation was conducted with CDK2 antibody in RPMI‐8226 and U266 cells, respectively. Western blot analysis of altH19 and CDK2 binding. (E) In vitro purification of GST‐CDK2 and altH19 protein, and GST‐pull down assay to examine the binding between CDK2 and altH19. (F, G) Immunoprecipitation using p‐CDK2 antibody in RPMI‐8226 and U266 cells, followed by western blot analysis of altH19 and p‐CDK2 binding. (H) In vitro induction and purification of GST‐CDK2 at 30°C (mainly in the phosphorylated form), and GST‐pull down assay to examine the binding between p‐CDK2 and altH19. (I) Immunofluorescence staining of RPMI‐8226 and U266 cells using altH19 and p‐CDK2 antibodies. DAPI indicating the cell nucleus. Scale bar: 5 μm. Experiments were repeated at least three times.

To explore the precise mechanism through which altH19 influences DNA replication and mitosis, we performed Flag affinity purification and mass spectrometry analysis using altH19‐overexpressing and vector control cells. GO enrichment analysis revealed that altH19 primarily participated in biological processes such as chromosome organisation regulation, DNA replication and chromosome remodelling. Interestingly, CDK2 was implicated in all of these processes (Figure S4A), suggesting a potential interaction between CDK2 and altH19. Subsequently, we conducted immunoprecipitation using an altH19 antibody in MM cells. The results confirmed that altH19 bound to CDK2 in both RPMI‐8226 and U266 cell lines (Figure 6A,B). To further validate this interaction, we performed immunoprecipitation with a CDK2 antibody, which specifically binds to CDK2 protein. This experiment verified that CDK2 interacted with altH19 in myeloma cells (Figure 6C,D). To determine if this interaction was direct, we expressed GST‐tagged CDK2 in *E. coli* BL21 cells and purified the GST‐CDK2 protein at 25°C. We then purified altH19 from eukaryotic cells using an altH19 antibody. GST‐pull down assays revealed no direct binding between altH19 and CDK2 (Figure 6E), suggesting that the interaction is mediated by other partner proteins or alternative forms of CDK2.

Previous studies have demonstrated that CDK2's biological function is activated upon phosphorylation and interaction with cyclins. Therefore, we performed immunoprecipitation using an antibody specific for phosphorylated Threonine 160 on CDK2 (p‐CDK2) and confirmed that p‐CDK2 interacted with altH19 in RPMI‐8226 and U266 cells (Figure 6F,G). These finds support our hypothesis that phosphorylation of CDK2 exposed specific binding sites that enable interaction with altH19. Abbas et al. [28] previously reported that CDK2 phosphorylation is minimal when expressed in *E. coli* at 25°C but significantly increases when induced at 30°C. Following their protocol, we re‐induced CDK2 expression at 30°C and purified the phosphorylated CDK2 protein (Figure S4B). We further validated the phosphorylation efficiency using a p‐CDK2 (Thr 160) antibody (Figure S4C). GST‐pull down assay using purified p‐CDK2 and altH19 confirmed a direct interaction (Figure 6H). Additionally, we purified GST‐CDK2 protein from eukaryotic HEK293T cells, where CDK2 exists in both phosphorylated and non‐phosphorylated forms. GST‐pull down assay further confirmed that altH19 interacted with phosphorylated CDK2 directly (Figure S4D). To further confirm this finding, we performed immunofluorescence staining of RPMI‐8226 and U266 cells using antibodies against altH19 and p‐CDK2. Confocal microscopy revealed co‐localization of altH19 and p‐CDK2 primarily in the nucleus (Figure 6I). In conclusion, our findings indicate that the micropeptide altH19 directly binds to phosphorylated CDK2 at threonine 160.

### 3.6 altH19 Promotes CDK2 Phosphorylation and Activates RB Signalling Pathways

**FIGURE 7:**
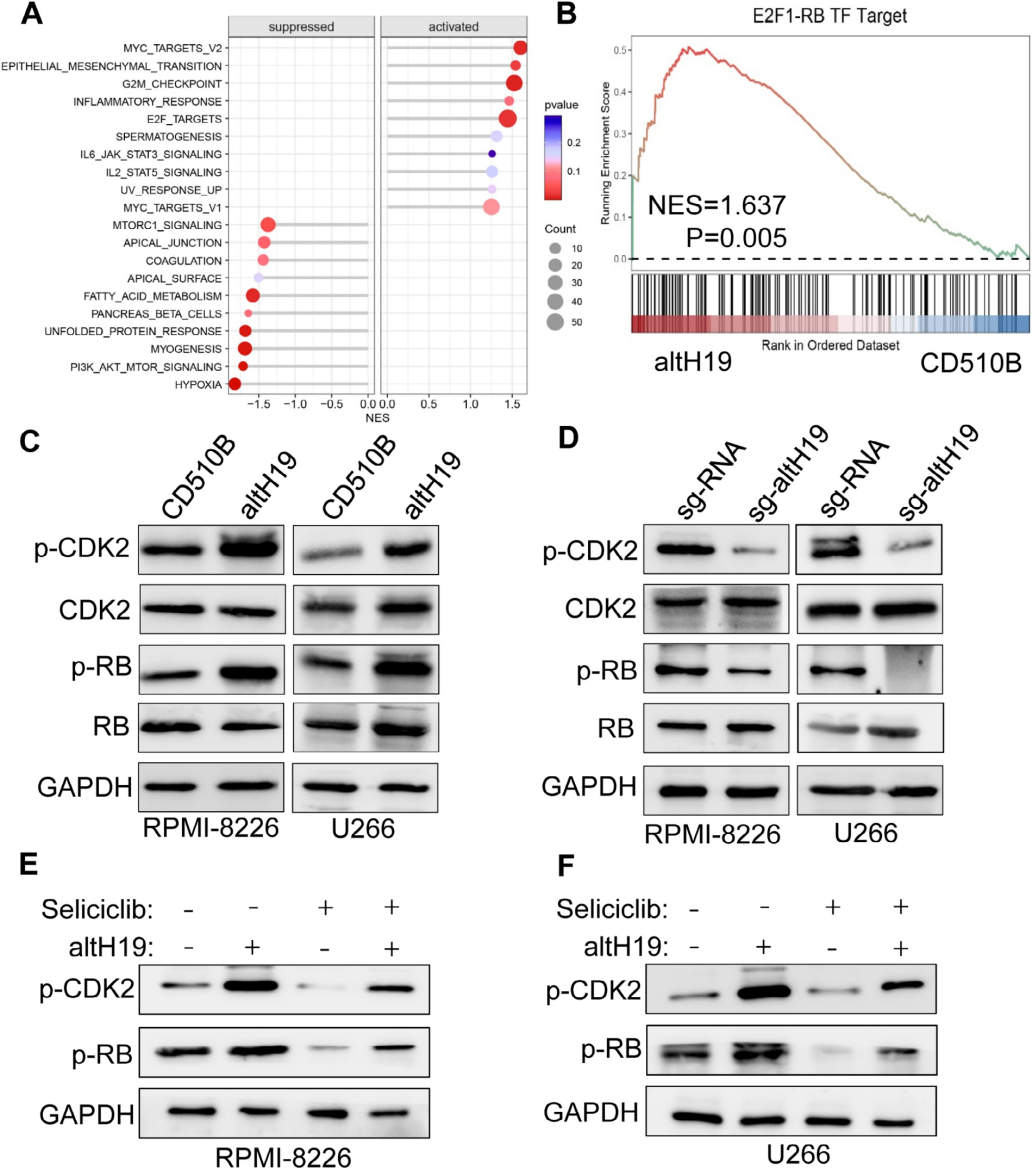
altH19 activates CDK2 phosphorylation and RB signalling pathways. (A) Proteomics was performed using RPMI‐8226‐CD510B and ‐altH19 cells, with a bubble chart showing the differential suppressed and activated gene sets. (B) GSEA analysis of the enrichment of altH19‐activated E2F1‐RB target gene. (C) Western blot analysis of p‐CDK2 (Thr160), CDK2, p‐RB (Ser780) and RB expression in altH19‐overexpessing RPMI‐8226 and U266 cells. (D) Western blot analysis of p‐CDK2 (Thr160), CDK2, p‐RB (Ser780) and RB expression in altH19‐knockout RPMI‐8226 and U266 cells. (E, F) Western blot analysis of p‐CDK2 and p‐RB expression in altH19‐overexpessing RPMI‐8226 and U266 cells treated with Seliciclib or not. All western blots were performed at least three times.

**FIGURE 8:**
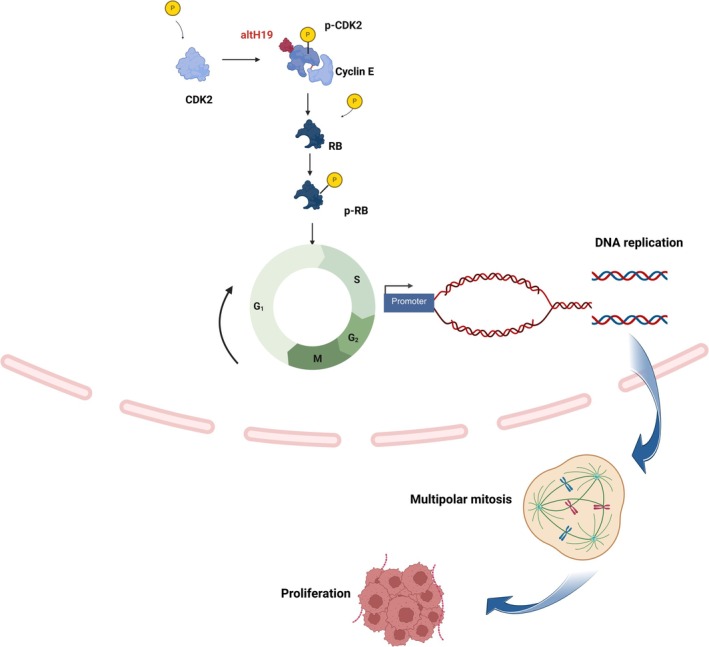
Schema diagram of altH19‐mediated myeloma progression. The micropeptide altH19 interacts with phosphorylated CDK2 (p‐CDK2), subsequently activating CDK2 phosphorylation. This enhances the downstream E2F1 target gene RB activity, which accelerates DNA replication, ultimately leading to rapid myeloma cell mitosis and proliferation.

We further explored how altH19 binding to p‐CDK2 drove its biological functions by performing proteomics on altH19‐overexpressing myeloma cells. As shown in Figure S4E, GO enrichment analysis demonstrated that altH19 overexpression was positively correlated with multiple biological processes, including mitotic cell cycle transition, DNA replication, cell cycle checkpoint signalling and mitotic nuclear division. GSEA analysis also revealed gene sets that were either suppressed or activated by altH19 (Figure 7A). Among these, we found that the E2F1‐RB target gene was enriched in upregulated gene sets in the altH19‐overexpressing group (Figure 7B), suggesting altH19 may activate RB pathway. But altH19 did not affect RB mRNA expression (Figure S4F,G). To further investigate this, we performed western blot analysis to examine the expression levels of CDK2 and its downstream target RB protein in both control and altH19‐overexpressing myeloma cells. The results showed that while the total protein levels of CDK2 and RB remained unchanged, the phosphorylation levels of both proteins were significantly elevated in the presence of altH19 (Figure 7C). Conversely, altH19 knockout led to a decrease in the phosphorylation levels of both CDK2 and RB, while the total protein levels of these molecules remained unaffected (Figure 7D). These results further support the notion that altH19 promotes CDK2 activation and consequently enhances RB phosphorylation. To validate this observation, we treated both control and altH19‐overexpressing cells with selective CDK2 inhibitor seliciclib. Western blot analysis showed that CDK2 and RB phosphorylation were significantly elevated upon altH19 overexpression but were effectively suppressed by seliciclib. Interestingly, even in the presence of seliciclib, altH19 overexpression partially reversed the inhibition of CDK2 and RB phosphorylation (Figure 7E,F). This suggests that altH19 plays a role in sustaining CDK2 enzymatic activity, which subsequently activates RB signalling. Taken together, these findings indicate that altH19 plays a crucial role in driving the CDK2‐RB signalling axis by enhancing CDK2 phosphorylation, thereby promoting cell cycle progression and the proliferation of MM cells (Figure 8).

## 4 Discussion

LncRNAs are transcripts longer than 200 nucleotides that were traditionally regarded as non‐coding byproducts of transcription. However, in recent years, numerous studies have highlighted the existence of stable, functional small peptides, also known as micropeptides, that are translated from lncRNAs [29]. This suggests that many functional micropeptides derived from lncRNAs remain undiscovered and unexplored. LncRNA H19 was the first imprinted gene identified, and it is dysregulated in various cancers, playing a crucial role in cancer progression and metastasis [30, 31, 32]. Therefore, H19 is considered a biomarker for cancer and a potential therapeutic target for a variety of human diseases. Traditionally, H19 was believed to be a non‐coding RNA without any protein‐coding capabilities. However, in this study, we identify a 97‐amino‐acid polypeptide derived from 5′ end of H19, which may represent an altORF of the H19 transcript. We have named this micropeptide altH19. Through multi‐omics experiments, we provide preliminary evidence suggesting that altH19 plays a role in biological functions associated with myeloma progression.

MM is a hematologic malignancy characterised by malignant proliferation of plasma cells, yet it remains incurable due to frequent relapse and drug resistance. Despite advances, the detailed mechanisms underlying myeloma development remain largely unexplored. In this study, we identified micropeptide altH19 as a key factor promoting myeloma cell proliferation by enhancing DNA replication speed. Transcriptomic and proteomic analyses revealed that CDK2 plays a central role in cell cycle transition, DNA replication, mitosis and chromatin remodelling in altH19‐overexpressing group. Further studies indicated that altH19 binds to phosphorylated CDK2 at threonine 160 directly. Previous studies have shown that CDK2 requires binding to various cyclins to initiate DNA replication [33]. Whether altH19 activates CDK2 phosphorylation through the participation of cyclin E or cyclin A remains an important question for future research. Additionally, CDK2 undergoes conformational changes upon phosphorylation by CAK kinase, which facilitates cell cycle progression [34]. While our study confirms that altH19 enhances phosphorylated CDK2 expression, the role of CAK kinase in this process remain unknown. We also employed the selective CDK2 inhibitor seliciclib to disturb CDK2 phosphorylation and observed that altH19 could partially reverse this inhibition in myeloma cells. This finding suggest that altH19 may interfere with the anti‐tumour effects of seliciclib, highlighting its potential as therapeutic target in overcoming resistance to CDK2 inhibitors.

Our data also demonstrated that altH19 accelerates the transition from early to mid‐late stages of DNA replication, significantly increasing the proliferation rate of myeloma cells. Additionally, altH19 promotes myeloma cell mitosis, with a higher proportion of cells undergoing multipolar division in altH19‐overexpressing cell lines. Multipolar division has been linked to chromosomal instability in various cancers [35, 36], and chromosomal instability is a key factor driving myeloma progression [37]. In our study, altH19 may contribute to chromosomal instability of myeloma cells by enhancing DNA replication efficiency and promoting multipolar mitosis. However, it remains unclear whether these processes lead to DNA damage and how altH19 might avoid immune checkpoint regulation during cell cycle. These questions warrant further investigation in future studies. Given the high expression of altH19 in myeloma patients and its role in regulating DNA replication and mitosis, altH19 is a promising therapeutic target. Combining altH19 knockout with specific CDK2 inhibitors offers a novel approach to improve MM therapy and warrants further exploration for targeted treatments.

## Author Contributions

**Yaxin Zhang:** writing original draft and investigation. **Wenjing Li**, **Jiwei Mao** and **Xiaodan Zhou:** investigation. **Xu Cao:** software, resources. **Linlin Liu:** review and editing, funding acquisition. **Ruosi Yao:** writing original draft, funding acquisition.

## Consent

All co‐authors agree to publication.

## Conflicts of Interest

The authors declare no conflicts of interest.

## Supporting information


**Data S1.** Supporting Information.

## Data Availability

Data sharing not applicable to this article as no datasets were generated or analysed during the current study.
