# Supplementary material for: LncRNA H19‐Encoded Micropeptide altH19 Promotes DNA Replication and Mitosis in Myeloma Cells by Enhancing the Phosphorylation of CDK2 at Threonine 160
Source: Cell Prolif. 2025 Jun 27;59(2):e70089. doi: 10.1111/cpr.70089 (PMC12877954; doi:10.1111/cpr.70089)
Supplement: Supplementary file 1 — Data S1. Supporting Information. [file CPR-59-e70089-s001.docx]

**
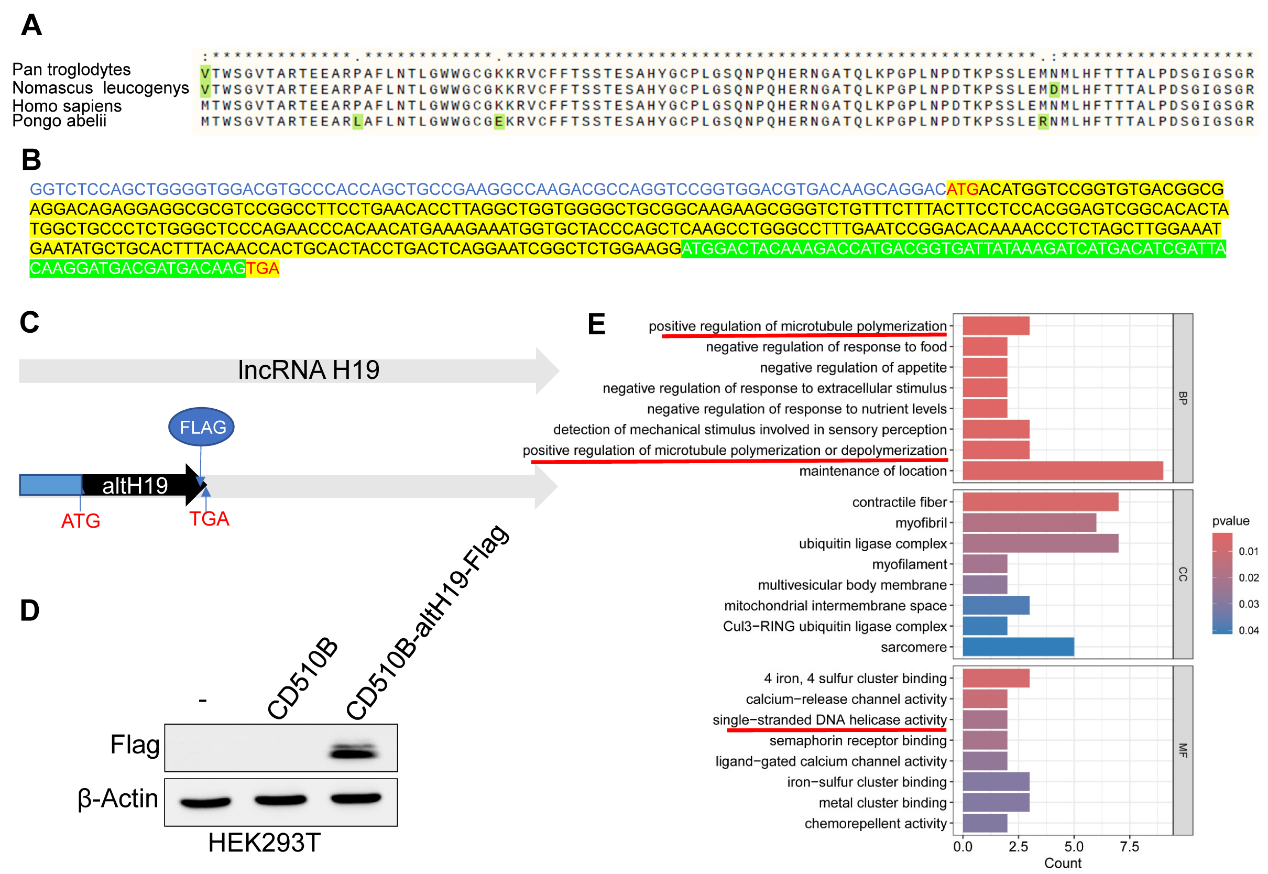
**

**Fig.S1:** A. Alignment of altH19 micropeptide sequences from a variety of mammals. Amino acid identity is indicated by asterisks. B. The cDNA sequence constructed in CD510B vector. C. Architecture of altH19 discovered on lncRNA H19 transcript. D. CD510B-altH19-Flag was transfected into HEK293T cells transiently, WB analysis of Flag tag expression. E. Transcriptome sequencing and GO enrichment analysis.


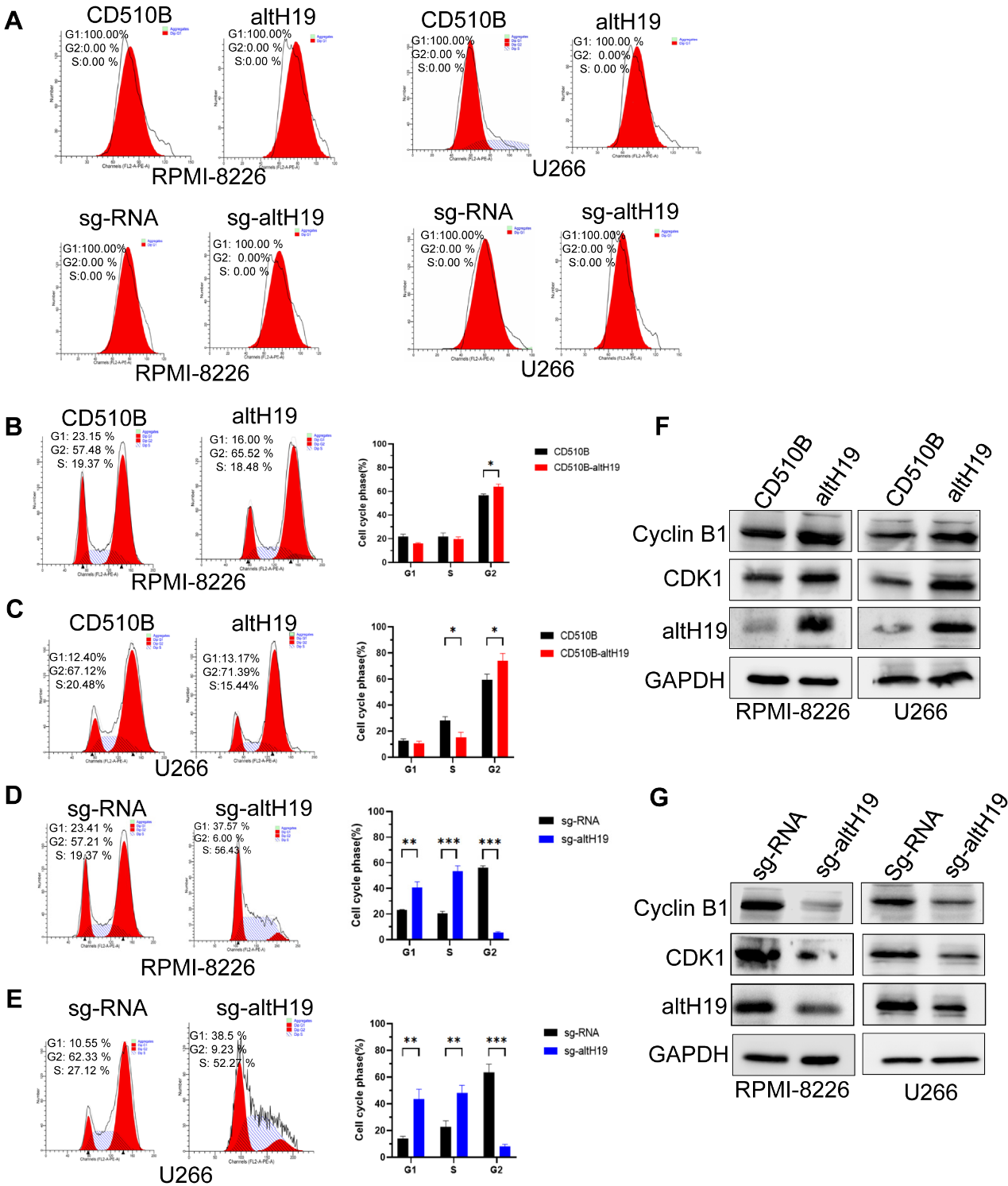


**Fig.S2:** A. The wild-type, altH19-overexpressing or altH19-knockout cells were conducted double synchronization treatment using thymidine and flow cytometry analysis of the proportion of G1 phase. B-C. After synchronization, flow cytometry analysis of subcellular distribution in altH19-overexpressing cells. D-E. After synchronization, flow cytometry analysis of subcellular distribution in altH19-knocking out cells. F-G. Western blot analysis of Cyclin B1, CDK1 and altH19 in altH19-overexpressing or knocking out cells. Experiments were conducted three times. mean ± SD. **, P<0.01; ***, P<0.001.


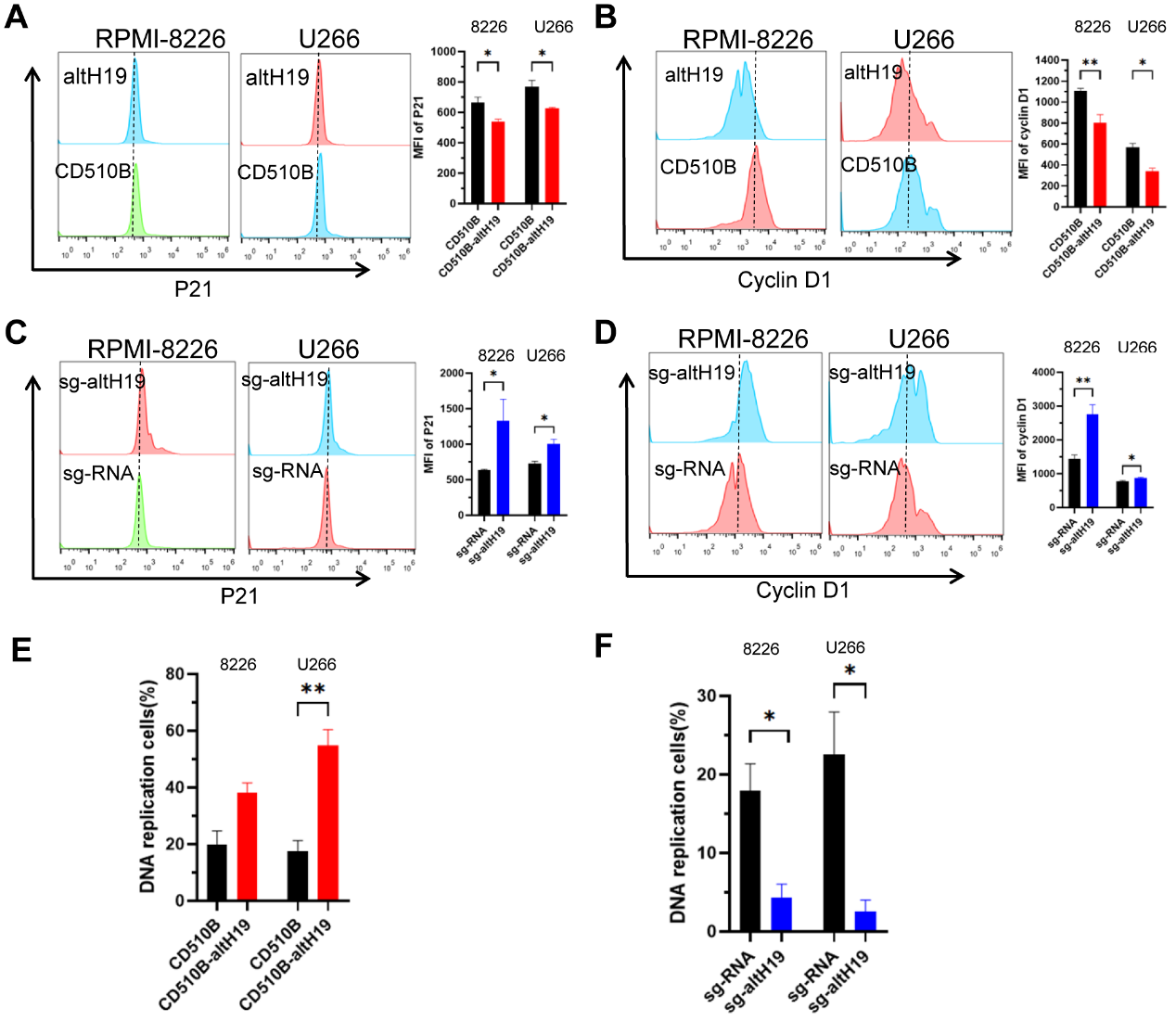


**Fig.S3:** A-B. P21 and Cyclin D1 were labeled, respectively. Flow cytometry analysis of mean fluorescent intensity in altH19-overexpressing RPMI-8226 and U266 cells. C-D. P21 and Cyclin D1 were labeled, respectively. Flow cytometry analysis of mean fluorescent intensity in altH19-knocking out RPMI-8226 and U266 cells. E-F. Indicated cells were stained using EdU after double synchronization. Immunofluorescence and histogram analysis the percent of DNA replication cells. Experiments were conducted three times. mean ± SD. *, P<0.05; **, P<0.01.


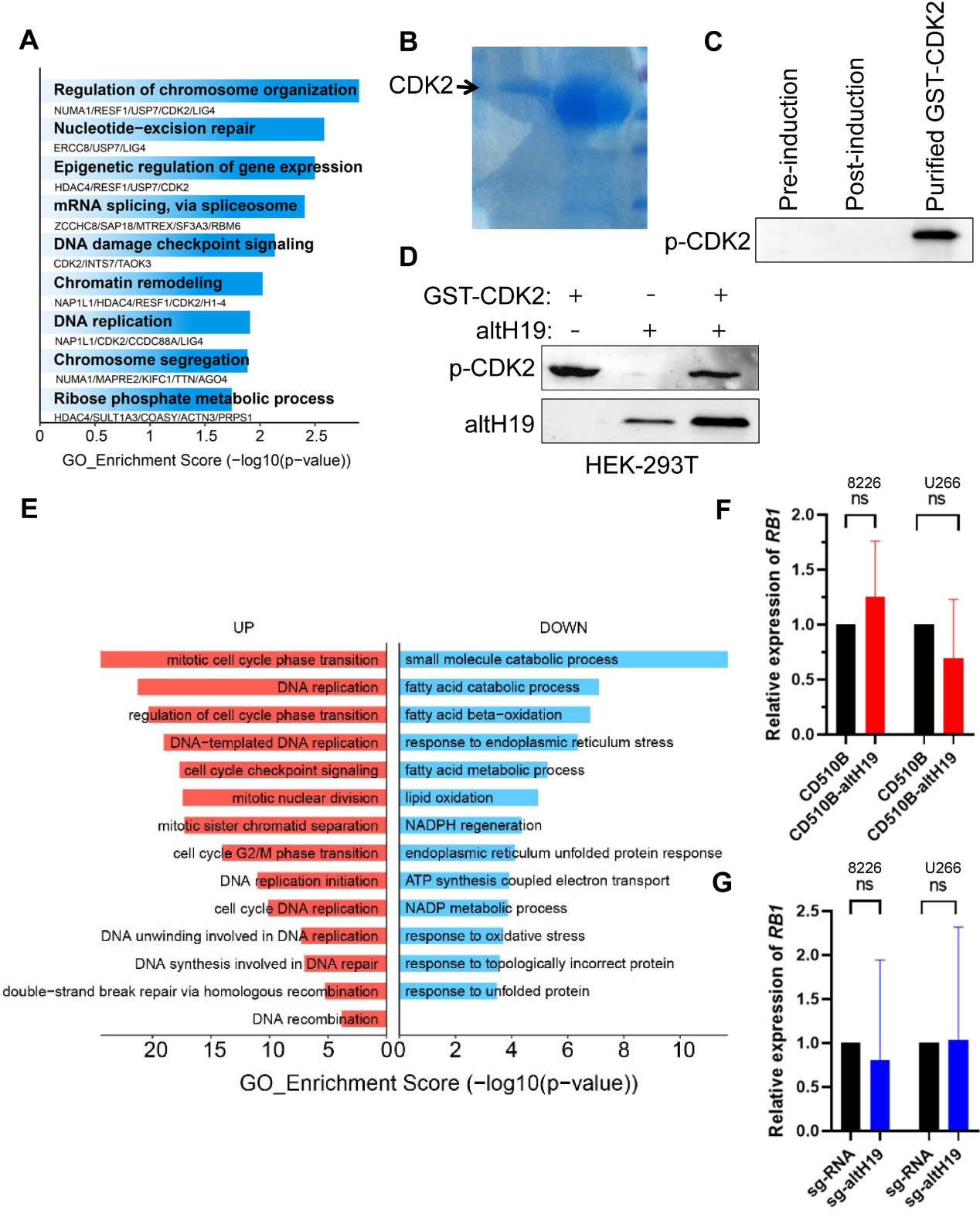


**Fig.S4:** A. Proteomics and GO enrichment analysis of biological processes regulated by altH19. B. GST-CDK2 protein was purified and stained using coomassie brilliant blue. C. GST-CDK2 was induced at 30°C and purified, western blot analysis of p-CDK2 protein at different stage. D. pcDNA3.1-GST-CDK2 plasmids were transfected into HEK293T cells, and GST-CDK2 protein was purified. GST pull down assay the interaction between altH19 and p-CDK2. E. GO enrichment analysis the correlation between altH19 and multiple biological processes. F. Q-PCR analysis of RB1 mRNA level in RPMI-8226 and U266 cells overexpressing altH19. G. Q-PCR analysis of RB1 mRNA level in RPMI-8226 and U266 cells with altH19 knockout.
